# Degradation of Axial Ultimate Load-Bearing Capacity of Circular Thin-Walled Concrete-Filled Steel Tubular Stub Columns after Corrosion

**DOI:** 10.3390/ma13030795

**Published:** 2020-02-10

**Authors:** Fengjie Zhang, Junwu Xia, Guo Li, Zhen Guo, Hongfei Chang, Kejin Wang

**Affiliations:** 1 Jiangsu Key Laboratory of Environmental Impact and Structural Safety in Engineering, China University of Mining and Technology, Xuzhou 221116, China; 2 Jiangsu Collaborative Innovation Center for Building Energy Saving and Construction Technology, Jiangsu Vocational Institute of Architectural Technology, Xuzhou 221116, China; 3 Department of Civil, Construction & Environmental Engineering, Iowa State University, Ames, IA 50011, USA

**Keywords:** axial ultimate load-bearing capacity, circular thin-walled CFST stub column, corrosion ratio, confinement coefficient, failure mode

## Abstract

This work aimed to investigate the effects of steel tube corrosion on the axial ultimate load-bearing capacity (AULC) of circular thin-walled concrete-filled steel tubular (CFST) members. Circular thin-walled CFST stub column specimens were made of steel tubes with various wall-thicknesses. These CFST column specimens were subjected to an accelerated corrosion test, where the steel tubes were corroded to different degrees of corrosion. Then, these CFST specimens with corroded steel tubes experienced an axial static loading test. Results show that the failure patterns of circular thin-walled CFST stub columns with corroded steel tubes are different from those of the counterpart CFST columns with ordinary wall-thickness steel tubes, which is a typical failure mode of shear bulging with slight local outward buckling. The ultimate strength and plastic deformation capacity of the CFST specimens decreased with the increasing degree of steel corrosion. The failure modes of the specimens still belonged to ductile failure because of the confinement of outer steel tube. The degree of steel tube corrosion, diameter-to-thickness ratio, and confinement coefficient had substantial influences on the AULC and the ultimate compressive strength of circular thin-walled CFST stub columns. A simple AULC prediction model for corroded circular thin-walled CFST stub columns was presented through the regression of the experimental data and parameter analysis.

## 1. Introduction

Concrete-filled steel tubes (CFSTs) are new composite materials produced by filling steel tubes with concrete. Their working principle is that the stability of the steel tube is immensely enhanced with the addition of hardened concrete. Thus, the axial ultimate load-bearing capacity (AULC) of outer steel tube under compression can be substantially improved, and the core concrete can exhibit a high compressive strength and ductility because of the confinement action of the outer steel tube [1,2]. CFSTs have been widely used in various civil engineering projects, such as multi and high-rise buildings, bridge structures, offshore platforms, large-span spatial structures, power transmission towers, and water-retaining walls because of their outstanding working performance [3,4,5,6,7,8]. In-depth studies have been conducted on the static and dynamic behavior, flexural behavior, fire resistance, and joint application of recycled concrete and CFST members [9,10,11,12,13]. Thin-walled or super thin-walled CFST members (with a confinement coefficient < 0.5 or steel tube wall-thickness < 3 mm) [14,15] can enhance the utilization efficiency of the steel and reduce steel consumption and engineering cost, thereby resulting in higher utilization values compared with ordinary CFST members [16,17,18]. However, the risk of AULC degradation caused by aggressive medium attacks also increases with the increasing applications of CFST structures in severe environments [19,20,21].

The steel tubes in CFST structures are more vulnerable to environmental corrosion compared with steel bars in reinforced concrete structures. Thus, the mechanical properties of corroded CFST members in the design of CFST structures are important, and have been investigated by many researchers [7,9,20,21,22,23,24]. Han et al. [7,20] and Hua et al. [9] studied the mechanical properties of circular and square CFST stub column specimens under sustained load and chloride corrosion. They found that the corrosion degree and sustained load have remarkable influences on the ultimate strength and ductility of CFST specimens, and high sustained loads or corrosion degrees cause a high reduction of specimens’ AULC and ductility. The failure patterns of CFST short columns before or after corrosion are approximately the same, which is a ductile failure where the steel tube experiences local outward buckling. Gao et al. [21] conducted axial compression tests on circular CFST short columns after combined corrosion and freeze–thaw cycles. They observed that the ultimate strength reduction of CFST columns is approximately linear with the corrosion ratios and freeze–thaw cycles, but the steel tubes’ corrosion ratios and freeze–thaw cycles have little effect on the failure modes of CFST columns. Chen et al. [22] and Yuan et al. [23,24] investigated the seismic performance of circular and square CFST columns after simulated acid rain attack. They concluded that the steel tube’s corrosion not only leads to an evident reduction in the yield strength, elastic modulus, and tensile strain capacity of steel coupons, but also results in considerable deterioration in the AULC, ductility, and energy dissipation of the CFST columns. The seismic behavior of circular CFST columns is more sensitive to corrosion compared to square CFST columns.

These studies on corroded CFST members have mainly focused on CFST members with ordinary steel tube wall thicknesses. However, corrosion is more harmful to thin-walled CFST members than ordinary CFST members because of the thinner wall thickness of their steel tubes. Thin-walled CFST members have lower confinement factors compared with ordinary CFST members. Thus, the confinement action of the outer steel tube on core concrete becomes weak, thereby resulting in a low AULC and plastic deformation capacity [16,25,26]. The risk towards the mechanical properties of thin-walled CFST members after corrosion is higher than that of ordinary CFST members. However, no systematic studies have been conducted on the mechanical properties of corroded thin-walled CFST members to date. This study aimed to investigate the failure modes and the degradation characteristics of the AULC of circular thin-walled CFST stub columns made with steel tubes under different corrosion ratios. A simple AULC prediction model for corroded circular thin-walled CFST stub columns is presented.

## 2. Experimental

### 2.1. Raw Materials and Specimens’ Fabrication

**Figure 1:**
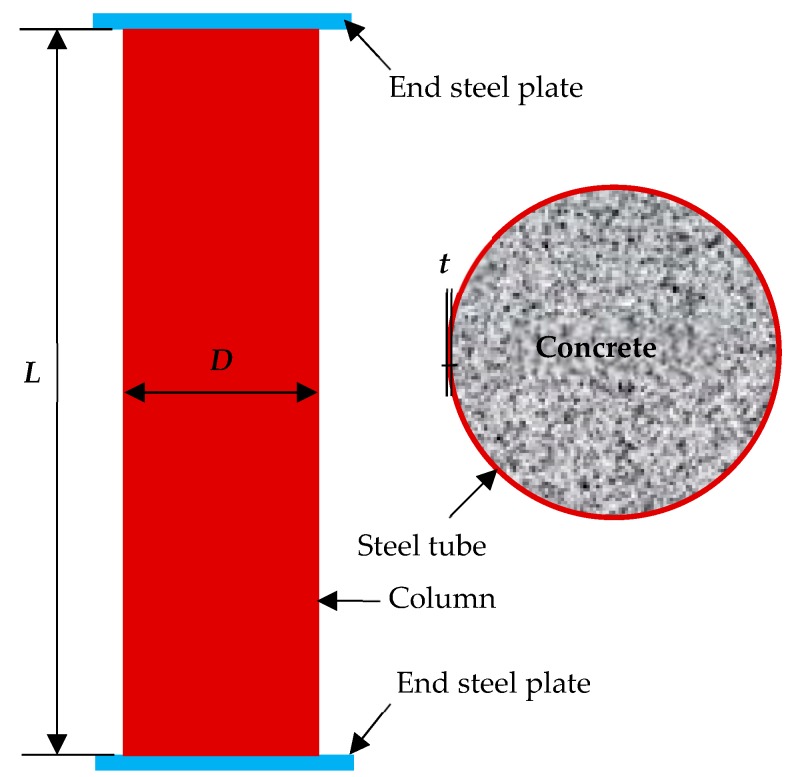
Schematic of a circular thin-walled CFST stub column.

Eleven circular CFST columns were designed on the basis of China’s technical specifications [14], where eight specimens underwent different corrosion degrees, and the three other specimens were used as the control group. All the circular CFST columns have an outer diameter of 139 mm and a height of 500 mm, which belong to a category of stub columns [14]. Their steel tubes have different wall thicknesses (0.92, 1.42, and 1.92 mm), corresponding to diameter-to-thickness ratios of 151.1, 97.9, and 72.4, respectively. A welded steel tube with a strength grade of Q235 was adopted; its measured coupon yield strength and elastic modulus were 238 MPa and 206 GPa, respectively. Core concrete with a strength grade of C30 was utilized, and its measured 28-day cube strength was 32.6 MPa. One end of the steel tube was sealed with a 200 × 200 × 10 mm^3^ steel plate prior to concrete casting, and the other end was sealed after concrete hardening for 28 days. Prior to sealing, the end surface of the core concrete and the outer steel tube were carefully polished to ensure close contact with the end steel plate. The details of the circular thin-walled CFST stub column are shown in Figure 1.

The mass corrosion ratio *β_0_* (Equation (1)) is generally adopted in expressing steel corrosion [21,27]. However, in the study of the corrosion of thin-walled steel tubes, the wall-thickness corrosion ratio *β* (Equation (2)) is more convenient than the mass corrosion ratio [7,20].

(1) $$\beta_{0}=\frac{m_{0}-m}{m_{0}}\times 100\%$$

(2) $$\beta =\frac{t_{0}-t}{t_{0}}\times 100\%$$

where *β_0_* and *β* are the mass corrosion ratio and the wall-thickness corrosion ratio (%), *m_0_* and *m* are the mass of a steel tube before and after corrosion (g); *t_0_* and *t* are the wall-thicknesses of a steel tube before and after corrosion (mm).

The mass corrosion and wall-thickness corrosion ratios of a steel tube were compared. Equation (1) is transformed into Equation (3).

(3) $$\beta_{0}=\frac{m_{0}-m}{m_{0}}=\frac{V_{0}-V}{V_{0}}=\frac{A_{0}-A}{A_{0}}=\frac{D\left(t_{0}-t\right)-{\left(t_{0}-t\right)}^{2}}{Dt_{0}-t_{0}^{2}}$$

where *V_0_* and *V* are the volume of a steel tube before and after corrosion (mm^3^), *A_0_* and *A* are the sectional area of a steel tube before and after corrosion (mm^2^); *D* is the original diameter of a steel tube before corrosion (mm).

The diameter-to-thickness ratio of a thin-walled CFST member is extremely high. Thus, the values of *t^2^*/*D* and (*t_0_* – *t*)^2^/*D* are negligible compared with wall-thickness *t* of a steel tube. Then, Equation (3) can be simplified into Equation (4).

(4) $$\beta_{0}=\frac{\left(t_{0}-t\right)-\frac{{\left(t_{0}-t\right)}^{2}}{D}}{t_{0}-\frac{t_{0}^{2}}{D}}\approx \frac{t_{0}-t}{t_{0}}=\beta$$

As shown in Equation (4), the two calculation methods for the steel tube’s corrosion degree are consistent for thin-walled CFST members. Taking a steel tube with a wall thickness of 0.92 mm and diameter of 139 mm as an example, the corresponding corrosion ratios of *β_0_* and *β* are 50.17% and 50%, respectively, when the 0.46 mm wall thickness of the steel tube is corroded, are approximately the same. Therefore, Equation (2) is used to calculate the corrosion ratios of steel tubes. An electrochemically accelerated corrosion method was adopted to achieve the required corrosion degrees for the steel tubes in a short time period [7,20]. The corroded mass of a steel tube was calculated on the basis of the designed corrosion degree using Faraday’s law and Equations (5)–(7) [9]. Then, the corresponding electric quantity (Q) for the required mass loss was calculated. The corrosion time *T* was obtained on the basis of constant current *I*. The detailed experimental plan for CFST specimens’ accelerated corrosion is listed in Table 1.

(5) $$\Delta m=\beta_{0}\times m_{0},$$

(6) $$Q=2\times \frac{\Delta m}{M}\times N_{A}\times e,$$

(7) $$T=\frac{Q}{I}$$

where Δ*m* is the mass loss of a corroded steel tube (g), *Q* is the required quantity of electricity I; *M* is the molar mass of iron (56 g/mol); *N_A_* is the Avogadro constant (6.02 × 10^23^/mol); *e* is the charge of an electron (1.602 × 10^−19^ C); *T* is the corrosion time required (s); *I* is the corrosion current intensity, taken l−2 mA/cm^2^.

**Figure 2:**
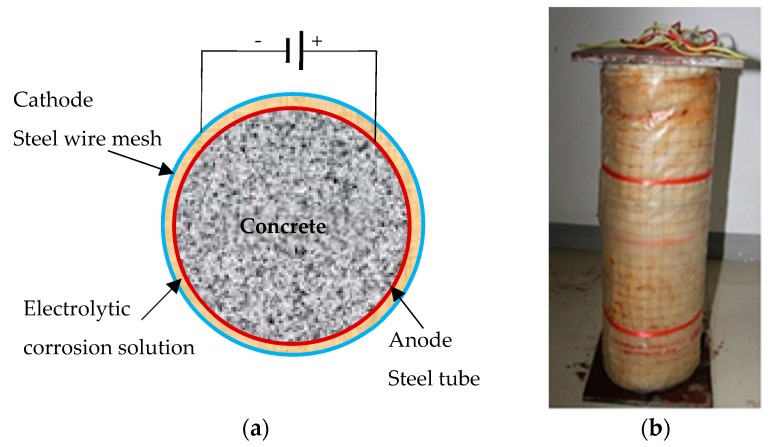
Schematic and photograph of accelerated corrosion on a CFST column. (**a**) The schematic; (**b**) a photograph.

During the electrochemically accelerated corrosion of CFST columns, a constant voltage power was used as the direct current (DC) supply, and the outer steel tube was connected to the anode of DC power. The steel tube surface was initially covered with a sponge soaked in 3% NaCl solution and then enveloped with a stainless steel wire mesh connected to the cathode of the DC power. In addition, plastic sheets were utilized to avoid moisture loss from the surfaces of the steel tubes. The steel plates at the two ends of the column were epoxy-coated to avoid corrosion. The schematic of accelerated corrosion on a CFST column and its photograph are presented in Figure 2. After the corrosion time calculated in Table 1 was reached, the external sponge and stainless steel wire mesh were removed, and the surfaces of these columns were cleaned. A micrometer was used to measure the residual diameter of the CFST column and, for each column, six residual diameters were measured along the column height. The averaged value of a CFST column’s residual diameters was used to calculate its actual corrosion degree, and then axial compression tests were conducted as the next step.

### 2.2. Experimental Procedure and Testing Parameters

Prior to the axial compression test, the surfaces of two of the end plates of a CFST column were scraped and polished, and the superficial impurities were removed to guarantee a uniform stress condition during loading. During the test, vertical displacement meters were installed at the two ends of the specimen to ensure axial loading, and transverse displacement meters were utilized to inspect the lateral bending of the specimen. Photo Information Technology [28] was used to record the CFST columns’ real-time images during the entire loading.

The theoretical AULC of a circular thin-walled CFST stub column before corrosion was calculated using Equation (8) [14].

(8) $$N_{0}=0.9A_{c}f_{c}\left(1+\alpha \theta \right),$$

(9) $$\theta =\frac{A_{s}f_{y}}{A_{c}f_{c}}$$

where *N_0_* is the theoretical AULC of a circular thin-walled CFST stub column (N); *θ* is the confinement coefficient of a CFST column; *α* is the coefficient relating to strength grade of core concrete; *A_c_* and *A_s_* are the cross sectional area of the core concrete and steel tube (mm^2^); *f_c_* and *f_y_* are the designed values of compressive strength of the core concrete and steel tube (Mpa).

**Figure 3:**
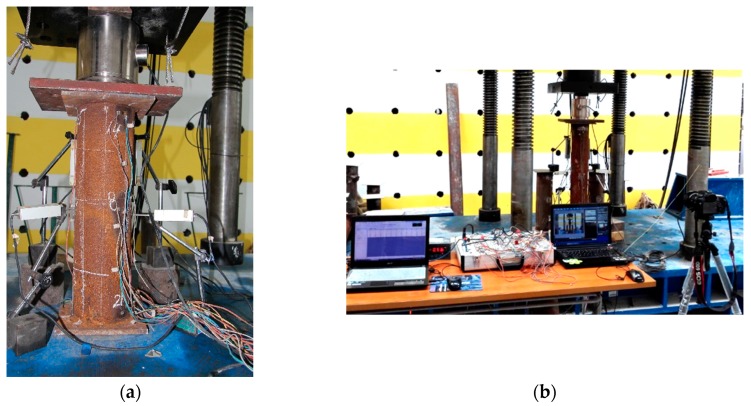
Photo of test setup and data acquisition devices. (**a**) The specimen; (**b**) the data acquisition device.

Axial loading was conducted using a 7000-kN hydraulic compression machine. The specimens were axially loaded using multistage loading. During the initial elastic deformation stage, 10% of the ultimate load *N_0_* was adopted, with 5% of the ultimate load *N_0_* being adopted during the plastic deformation stage. The loading at each stage lasted for 5 min. After the deformation became stable, the data were collected, and the next loading stage started. All the data were automatically collected by a computer-aided data acquisition system. The test setup and data acquisition devices are shown in Figure 3. 

## 3. Results

### 3.1. Curves of Loads Versus Displacement

**Figure 4:**
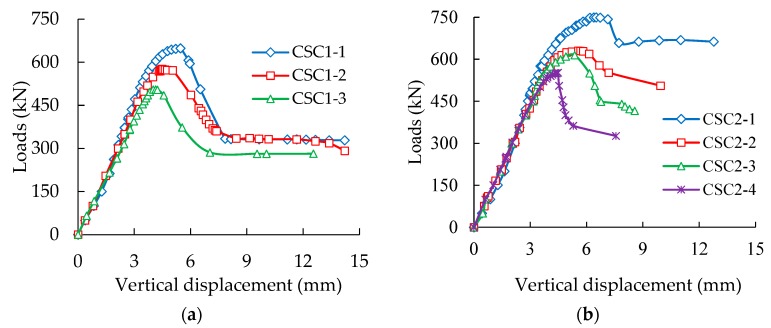

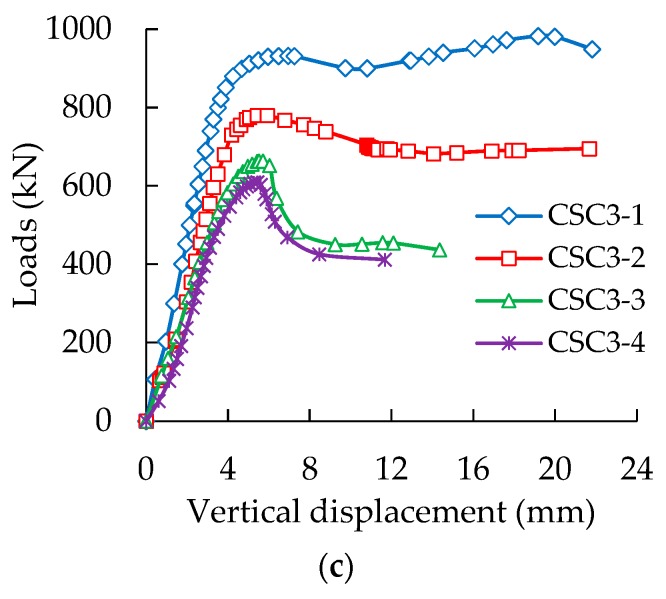
Load vertical displacement behavior of CFST specimens with different corrosion ratios. (**a**) *t* = 0.92 mm; (**b**) *t* = 1.42 mm; (**c**) *t* = 1.92 mm.

The development of loads with longitudinal displacements of the CFST specimens with different corrosion ratios was plotted, as shown in Figure 4. The load-displacement curves of all CFST columns can be divided into four stages, namely linear uprising, nonlinear uprising, nonlinear steep drop, and slow drop. During the initial stage, the load-displacement curves linearly increased, and no changes were found on the surfaces of the CFST columns. With the increase in load, the second stage of nonlinear uprising started, and steel scraps (rust attached on the surfaces of steel tubes) began to be removed, accompanied by a slight noise. The vertical displacement of the specimens rapidly increased, and bulge deformation started to occur at the midpoint of a column specimen with a continuous increase in load. The third stage of nonlinear steep drop started when the peak load of a CFST column was reached (accompanied by a clear, loud and brittle sound). The specimens rapidly deformed, and the load sharply decreased. Evident shear failure planes formed in the body of the CFST columns, and the last stage of slow drop started. During this period, the load gradually decreased, whereas the deformation rapidly increased until the complete failure of the specimens.

Subsequent observation indicated that the high wall thickness of a steel tube corresponded to the high AULC of a CFST stub column, whether the steel tubes were corroded or not. For each kind of CFST column, the AULC of a column and the corresponding longitudinal deformation decreased with an increase in steel tube corrosion ratios. This condition proves that the corrosion of the outer steel tube reduces the AULC and plastic deformation capacity of circular thin-walled CFST stub columns.

### 3.2. Failure Modes

**Figure 5:**
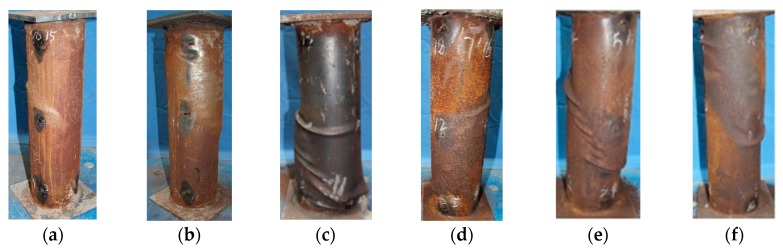
Photographs of CFST columns after failure. (**a**) CSC1-1; (**b**) CSC2-1; (**c**) CSC3-1; (**d**) CSC1-3; (**e**) CSC2-4; (**f**) CSC3-2.

Photographs of the typical column specimens after failure are shown in Figure 5. Compared with the failure modes of ordinary CFST stub columns (local outward buckling) and hollow steel tubes (outward and inward local bucking) under axial compression [7,20], these circular thin-walled CFST short columns exhibited a failure pattern of obvious shear bulge with slight local outward buckling, whether the steel tube was corroded or not. Evident shear deformation lines of approximately 45° were found on the steel tubes, which were consistent with the failure modes of CFST columns reported in the literature [21,29].

**Figure 6:**
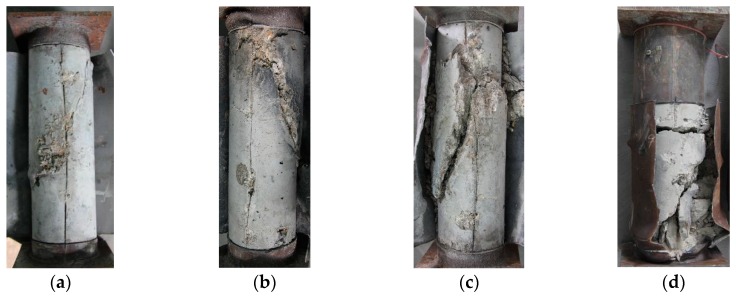
Typical photographs of the core concrete after failure. (**a**) CSC1-3; (**b**) CSC2-2; (**c**) CSC2-4; (**d**) CSC3-2.

After the compression tests on CFST columns, the outer steel tubes were removed, and the typical failure patterns of the core concrete were observed (Figure 6). The failure modes of the core concrete in 11 CFST columns were mainly shear failures (evident shear planes formed in specimens) accompanied by local concrete crushing. Some minor cracks were initially formed in the concrete under pressure. With the increase in load, the cracks grew, and the core concrete expanded. The development of concrete cracks was limited because of the restraint of the outer steel tube. A major crack was formed with the continuous increase in load, thereby causing core concrete fracture.

The stress condition of the core concrete in a CFST stub column belongs to the 3D stress conditions. The core concrete, under axial compression, produces lateral expansion, resulting in an outward compressive stress on the outer steel tube and generates radial tension stress inside the steel tube to balance it. The steel tube is in an elastic condition, and its deformation is small when the radial tension stress is relatively low. Therefore, the steel tube can produce sufficient restraint on the core concrete. The steel tube produces evident plastic deformation when its tension stress reaches its yield strength, and thereby cannot provide sufficient constraints on the core concrete. Chen et al. [29] and Chen et al. [30] indicated that the ultimate strength and failure modes of concrete under a triaxial stress state are closely related to the confining pressures. Low confining pressures correspond to the low axial ultimate strength of concrete, and the failure modes are close to the brittle shear failure of concrete. On the contrary, high confining pressures lead to the high axial ultimate strength of concrete, and the failure modes are close to the extrusion plastic failure of concrete. The thin-walled CFST stub columns used in this study had a thin wall thickness, and the wall thickness became even thinner after corrosion. Thus, the confining effects provided by the outer steel tube were limited, resulting in the shear failure of core concrete and the corresponding shear bulging of the outer steel tube. Although the core concrete failed in a sudden, brittle manner, such phenomena did not occur in the CFST columns. This condition is because the circular thin-walled CFST short columns have a certain plastic deformation capability, owing to the contribution of the outer steel tubes.

## 4. Discussion

### 4.1. Effect of Steel Tube’s Corrosion on Specimens’ Relative AULC

**Figure 7:**
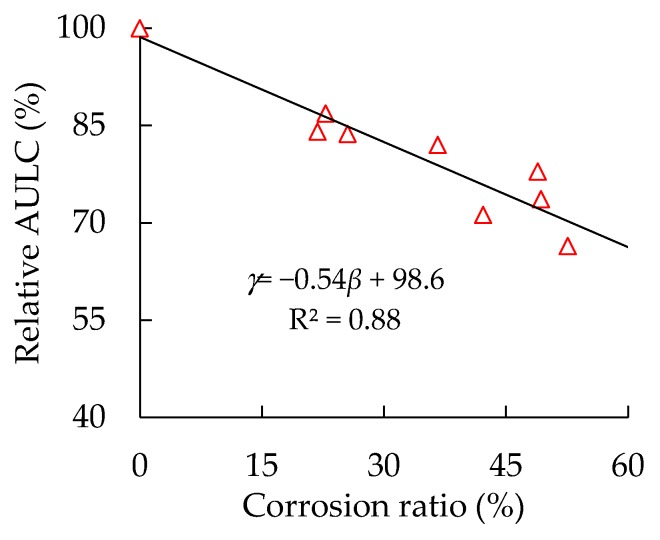
Relationship between specimens’ relative axial ultimate load-bearing capacity (AULC) and steel tube corrosion ratios.

The AULC of a CFST column is composed of the outer steel tube and the core concrete. The steel tube’s corrosion not only directly reduces the sectional area of steel tube parts, resulting in the reduction of their AULC, but also reduces the confinement of the steel tube on the core concrete. Accordingly, the confining coefficient and the AULC of the core concrete part are reduced [9,20]. To quantitatively study the influence of the corrosion ratios of steel tubes on the AULC of specimens, a new parameter *γ* of relative AULC is defined, as shown in Equation (10). The relationship between the specimens’ relative bearing capacity and their corrosion degrees is shown in Figure 7. The AULC of corroded circular thin-walled CFST stub columns is linearly related to their corrosion degrees, and the correlation coefficient R^2^ reaches 0.88. This finding indicates that the high corrosion degrees of the steel tube correspond to the low AULC of the CFST columns.

(10) $$\gamma =\frac{N_{u}}{N_{u0}}\times 100\%$$

where *γ* is the relative AULC of a corroded CFST column (%), *N_u0_* and *N_u_* are the measured AULC of a CFST column before and after the steel tubes’ corrosion (N).

As shown in Figure 7, although the reductions of the AULC of corroded CFST columns are proportional to the increase in the steel tube’s corrosion ratios, their change rates are different. For a CFST column with a wall thickness of 0.92 mm, the reductions in AULC are 13.1% and 22.1%, corresponding to the corrosion ratios of 22.8% and 48.9%. In other words, the reduction amplitude of the AULC is approximately half of the increase in the amplitude of the steel tube’s corrosion ratios. The decreasing rates of the AULC of corroded circular thin-walled CFST stub columns are lower than the increasing rates of the steel tube’s corrosion ratios. This condition is mainly because steel tubes can still have certain confining effects after corrosion, thereby utilizing the ultimate strength of the core concrete.

### 4.2. Effect of Diameter-to-Thickness Ratio on Specimens’ Ultimate Compressive Strength

**Figure 8:**
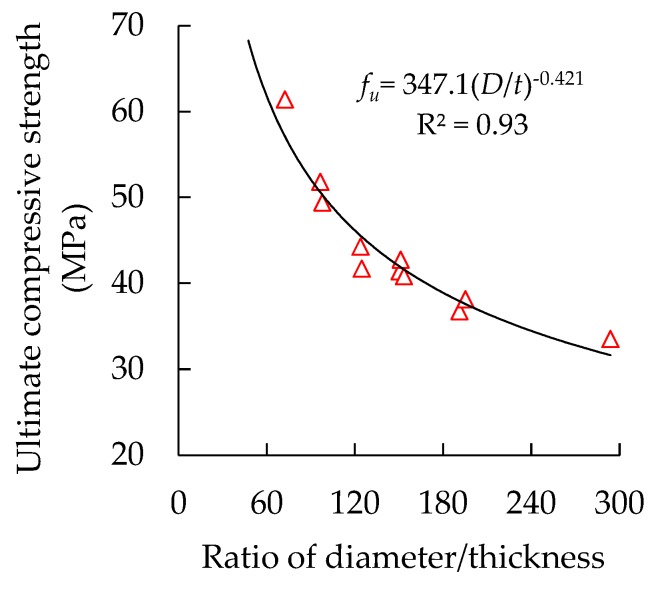
Relationship between specimens’ ultimate compressive strength and diameter/thickness ratios.

Diameter/thickness is an important parameter of a circular thin-walled CFST stub column, and has important influences on its AULC [20,21]. The actual diameter/thickness ratio of a CFST column changes with the corrosion of the steel tubes. Given that strength is a more normalized property of an axil compression member than load, the ultimate compressive strength *f_u_*, defined in Equation (11), was utilized instead of AULC. The ultimate compressive strength of corroded column specimens with their diameter-to-thickness ratios are plotted and presented in Figure 8, on the basis of the measured residual diameter of the steel tubes.

(11) $$f_{u}=\frac{N_{u}}{A_{u}}$$

where *f_u_* is the ultimate compressive strength of a corroded CFST column (MPa), *A_u_* is the measured sectional area of a CFST column after the steel tube’s corrosion (mm^2^).

The increase in the diameter/thickness ratios suggests that the sectional area of the steel tube decreases relative to the entire column sectional area. In particular, the restraint effect of the steel tube on the core concrete weakens, and the improvement effect of the material properties of the core concrete is reduced. With the increase in diameter/thickness ratios, the specimens’ ultimate compressive strength gradually decreases with a nonlinear curve style, and the correlation coefficient R^2^ reaches 0.93. The continuous increase in diameter-to-thickness ratios decreases the specimens’ ultimate compressive strength.

### 4.3. Effect of Confinement Coefficient on Specimens’ Ultimate Compressive Strength

**Figure 9:**
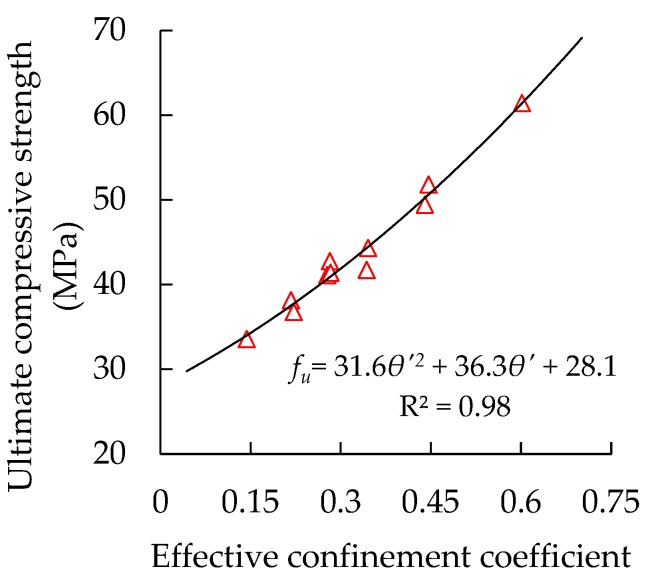
Relationship between specimens’ ultimate compressive strength and confinement factors.

The confinement coefficient refers to the restraint capacity of steel tubes on core concrete. The confinement factors decrease with the increase in the steel tube’s corrosion degree. The yield strength of the steel tubes remains unchanged before and after corrosion because of their uniform corrosion [9,20]. The new effective confinement coefficients were calculated (Equation (12) [7]) on the basis of the measured residual wall thickness of the steel tubes. The relationship between the specimens’ ultimate compressive strength and the effective confinement factors is plotted and shown in Figure 9.

(12) $$\theta^{\prime }=\frac{A_{s}^{\prime }f_{y}}{A_{c}f_{c}}$$

where *θ’* is the effective confinement coefficient of a CFST column after steel tube’s corrosion, *A_S_’* is the residual sectional area of a steel tube after corrosion.

A significant nonlinear growth relationship can be found between the specimens’ ultimate compressive strength and their effective confinement factors. The correlation coefficient R^2^ of the regression in Figure 9 reaches 0.98, which is higher than that of the regression of the steel tube’s corrosion ratio and diameter/thickness ratio, implying that the specimens’ ultimate compressive strength is highly relevant to their effective confinement factors. Thus, it can be used to establish an AULC prediction model for corroded circular thin-walled CFST stub columns.

### 4.4. Simple AULC Prediction Model for Corroded Specimens

The calculation methods for the AULC of the short CFST columns can be divided into two categories, where the first category is unified theory, that is, CFST is regarded as a composite material [15,31,32], and the second category is superposition theory, where the AULCs of the steel tubes and core concrete are separately calculated and added [3,10,16,17,33,34,35]. The designed value of the compressive strength of the CFST composite material must be known in order to calculate its AULC using unified theory. Such calculation methods are simple and clear, although obtaining the accurate strength of the composite material is difficult. Taking the CFST as the sum of two materials, the steel tube and core concrete separately have their advantages, while compensating with their disadvantages. A confinement coefficient is adopted to reflect the composite interaction between the steel tube and core concrete. This method directly expresses the combined action of the two materials and is consistent with the working mechanism of the CFST stub column as a compressive member under loads. Accurately evaluating the confinement action of the outer steel tube on core concrete is difficult.

Outward plastic deformation was generated in the steel tubes through axial compression tests of the corroded circular thin-walled CFST stub columns, indicating that the performance of the outer steel tubes can be fully utilized with the support of core concrete filling. The strength and ductility of the core concrete substantially improved because of the constraint of the outer steel tube. Combined with the experimental data and relevant parameter analysis in Section 4.3, superposition theory was adopted to calculate the AULC of the corroded circular thin-walled CFST stub columns using Equation (13) through nonlinear regression fitting.

(13) $$N_{p}=k_{1}N_{s}+k_{2}N_{c},$$

(14) $$k_{2}=1.516\theta^{\prime 2}+0.765\theta^{\prime }+1.236$$

where *N_p_* is the predicted AULC of a corroded circular thin-walled CFST column (N), *N_s_* and *N_c_* are the AULCs of corroded steel tube and core concrete, respectively, $N_{s}=A_{s}^{\prime }f_{y}$, $N_{c}=A_{c}f_{c}$, where the measured values of *A_s_’*, *A_c_*, *f_y_*, and *f_c_* should be used, *k_1_* and *k_2_* are the strength enhancement coefficients of the steel tube and core concrete with the support of the core concrete and the confining effects of the outer steel tube, respectively. The compressive strength of the steel tube can be fully utilized because of the filling and support of the core concrete. Therefore, *k_1_* can be set to one.

The correlation coefficient R^2^ of regression in Equation (13) is 0.95, indicating that the confidence of the model is extremely high. The effectiveness of Equations (13) and (15) in China’s specification [9] is compared in calculating the AULC of CFST stub columns, and the comparison of the calculation results using the two methods and experimental data is listed in Table 2.

(15) $$N_{p}=A_{sc}\left(1.212+B\theta +C\theta^{2}\right)f_{c},$$

*B* = 0.176*f_y_*/213 + 0.974, (16)

*C* = −0.104*f_c_*/14.4 + 0.031 (17)

where *A_sc_* is the sectional area of a CFST column, which is the sum of the sectional area of steel tube *A_s_’* and core concrete *A_c_*, *B* and *C* are the influencing coefficients of CFST columns’ cross-section shape on the confinement coefficients for a circular shape; *θ* is the confinement coefficient. Here, the effective confinement coefficient *θ‘* should be taken after the steel tube’s corrosion.

The fitting accuracy of Equation (13) is extremely high, whereas the results using Equation (15) have relatively larger errors compared with those of Equation (13), which are, on average, lower than the experimental data by 17%. Thus, Equation (13) can be applied to calculate the AULC of corroded circular thin-walled CFST stub columns. It is worth noting that the formula (Equation (13)) is only appropriate for corroded circular thin-walled CFST stub columns, and the mechanical properties of corroded thin-walled eccentric CFST stub columns or long CFST columns require further study.

## 5. Conclusions

Our conclusions, on the basis of experimental studies and theoretical analysis, are provided as follows:

- The corrosion of the outer steel tube reduces the AULC and plastic deformation ability of circular thin-walled CFST stub columns, and the high corrosion ratios cause high reduction. The degradation speeds of the AULC of circular thin-walled CFST stub columns are lower than the steel tube’s corrosion rates because of the composite interaction between the steel tube and the core concrete;
- The failure modes of axial circular thin-walled CFST stub columns are mainly shear bulging with slight local outward buckling, regardless of the degree of steel tube’s corrosion because of the limited restraint action of steel tubes on core concrete. Although the core concrete failed in a brittle shear failure mode, the failure mode of the column specimens still belongs to a ductile failure because of the confinement action of the outer steel tube;
- The AULC of a corroded circular thin-walled CFST stub column is closely related to the degree of corrosion of its steel tube, its diameter/thickness ratio and effective confinement coefficient, in which the correlation with the effective confinement factor is the highest. A simple model for predicting the AULC of corroded circular thin-walled CFST stub columns was presented through a regression analysis of the experimental data.

## Figures and Tables

**Table 1:** Experimental plan of concrete-filled steel tubular (CFST) specimens’ electrochemically accelerated corrosion.

No.	Item	*t_0_* /mm	*β* /%	*t_0_* – *t* /mm	Δ*m* /g	*I* /A	*T* /h
1	CSC1-1	0.92	0 (0)	0 (0)	0	0	0
2	CSC1-2	0.92	20 (22.8)	0.18 (0.21)	340.4	2.0	162.86
3	CSC1-3	0.92	50 (48.9)	0.46 (0.45)	851.1	2.0	407.15
4	CSC2-1	1.42	0 (0)	0 (0)	0	0	0
5	CSC2-2	1.42	20 (21.8)	0.28 (0.31)	510.7	2.0	244.29
6	CSC2-3	1.42	33.3 (36.6)	0.47 (0.52)	850.2	2.0	407.15
7	CSC2-4	1.42	50 (49.3)	0.71 (0.70)	1276.6	2.0	586.8
8	CSC3-1	1.92	0 (0)	0 (0)	0	0	0
9	CSC3-2	1.92	25 (25.5)	0.48 (0.49)	851.1	2.0	407.15
10	CSC3-3	1.92	40 (42.2)	0.77 (0.81)	1361.8	2.0	651.44
11	CSC3-4	1.92	50 (52.6)	0.96 (1.01)	1702.2	2.0	814.30

Note: the figures outside and inside parentheses are the calculated and measured corrosion ratio and wall-thickness reduction of a steel tube, respectively.

**Table 2:** Comparison of the calculated results and experimental data of specimens’ AULC.

Item	Experimental Data/kN	Prediction 1/kN	Error 1/%	Prediction 2/kN	Error 2/%
CSC 1-1	648.8	624.3	3.8	529.6	−18.4
CSC 1-2	577.2	569.4	1.4	501.8	−13.1
CSC 1-3	505.6	512.0	−1.3	470.1	−7.0
CSC 2-1	749.7	765.2	−2.1	588.3	−21.5
CSC 2-2	630.2	670.4	−6.4	547.7	−13.1
CSC 2-3	619.9	611.7	1.3	520.1	−16.1
CSC 2-4	552.2	564.9	−2.3	496.5	−10.1
CSC 3-1	931.9	930.7	0.1	645.9	−30.7
CSC 3-2	780.4	760.6	2.5	582.3	−25.4
CSC 3-3	664.4	662.9	0.2	540.7	−18.6
CSC 3-4	619.1	607.2	1.9	514.7	−16.9

Note: Prediction 1 results are obtained using Equation (13), whereas Prediction 2 results are obtained using Equation (15).
